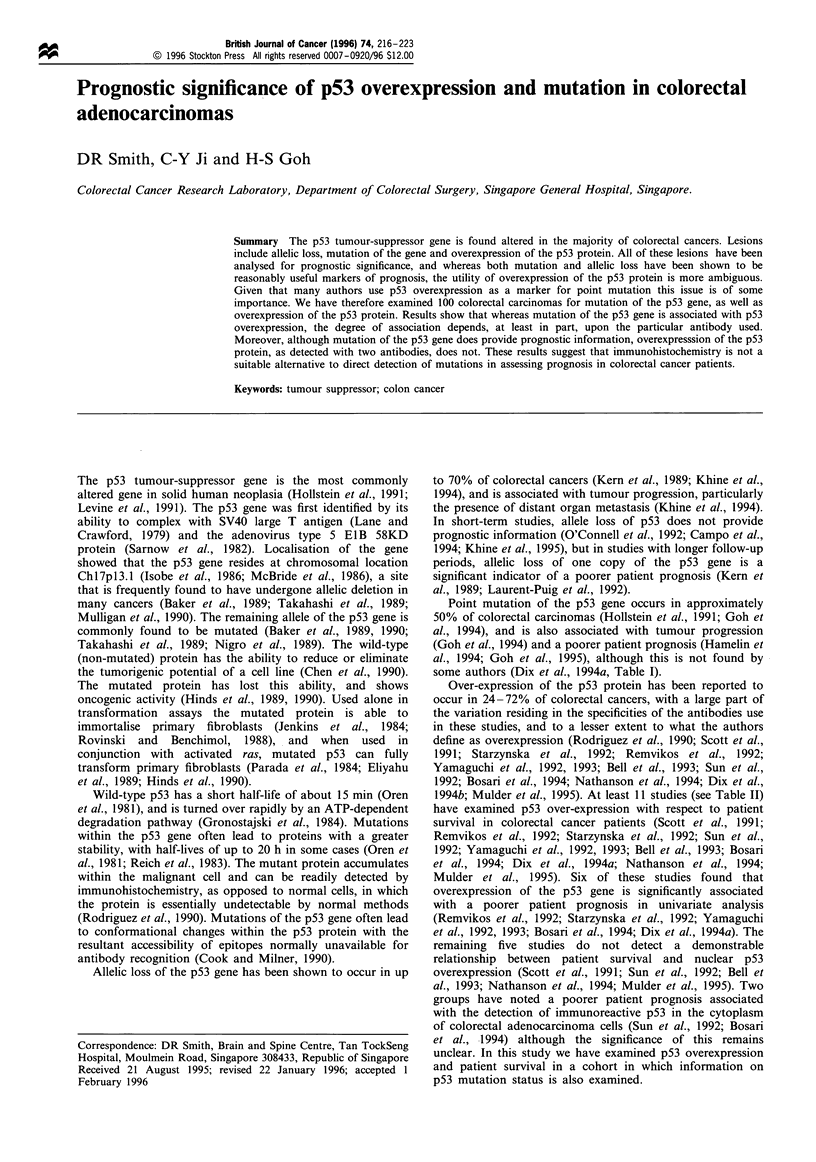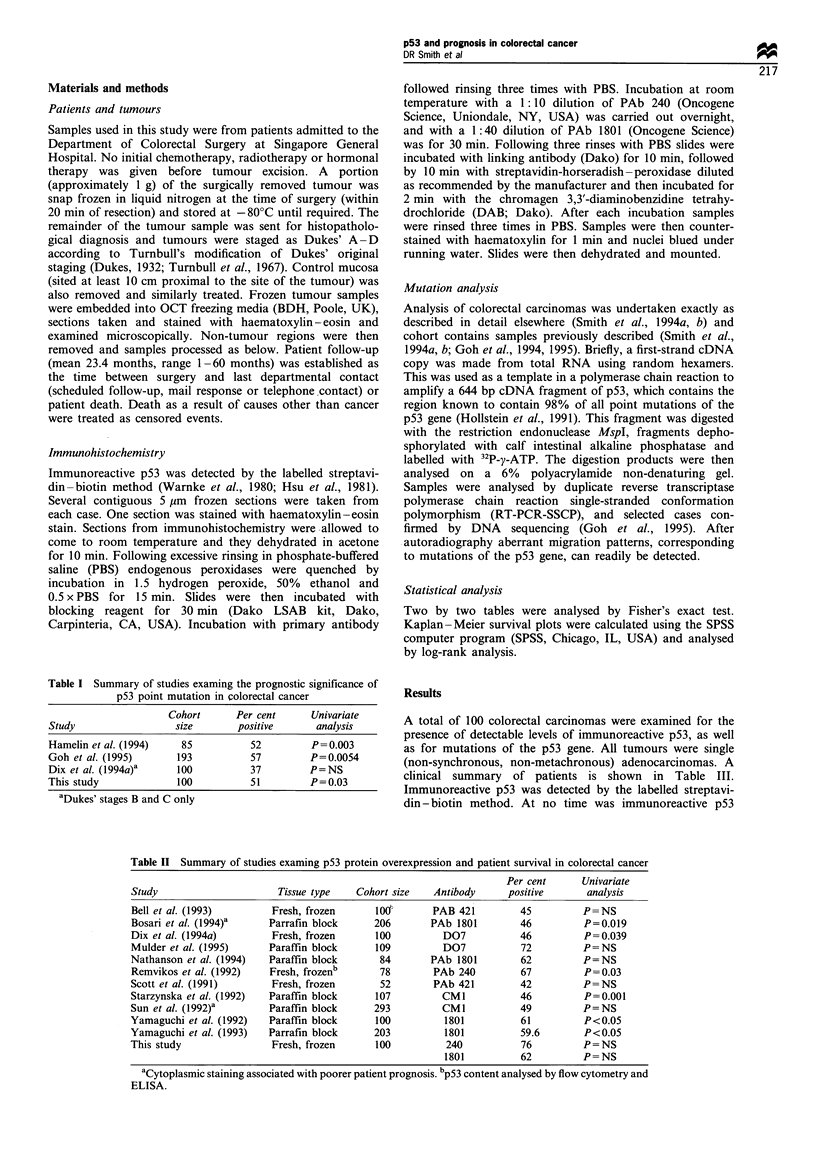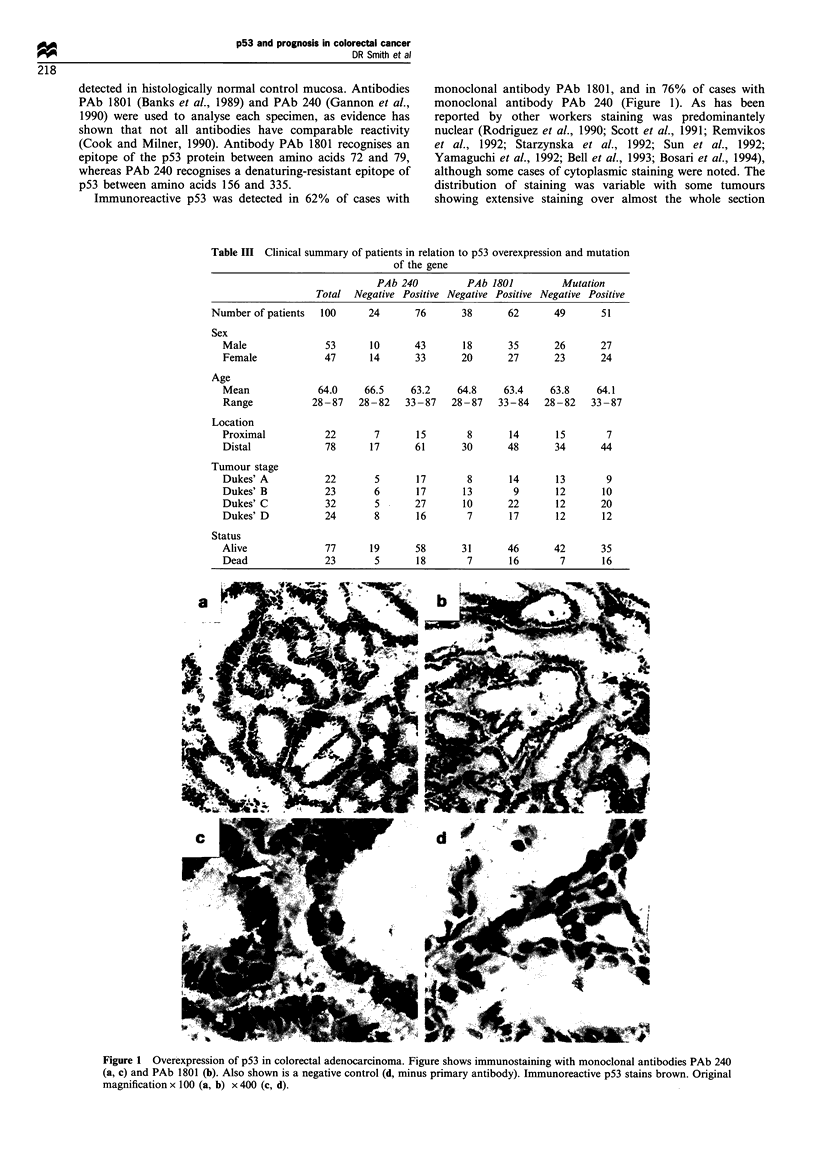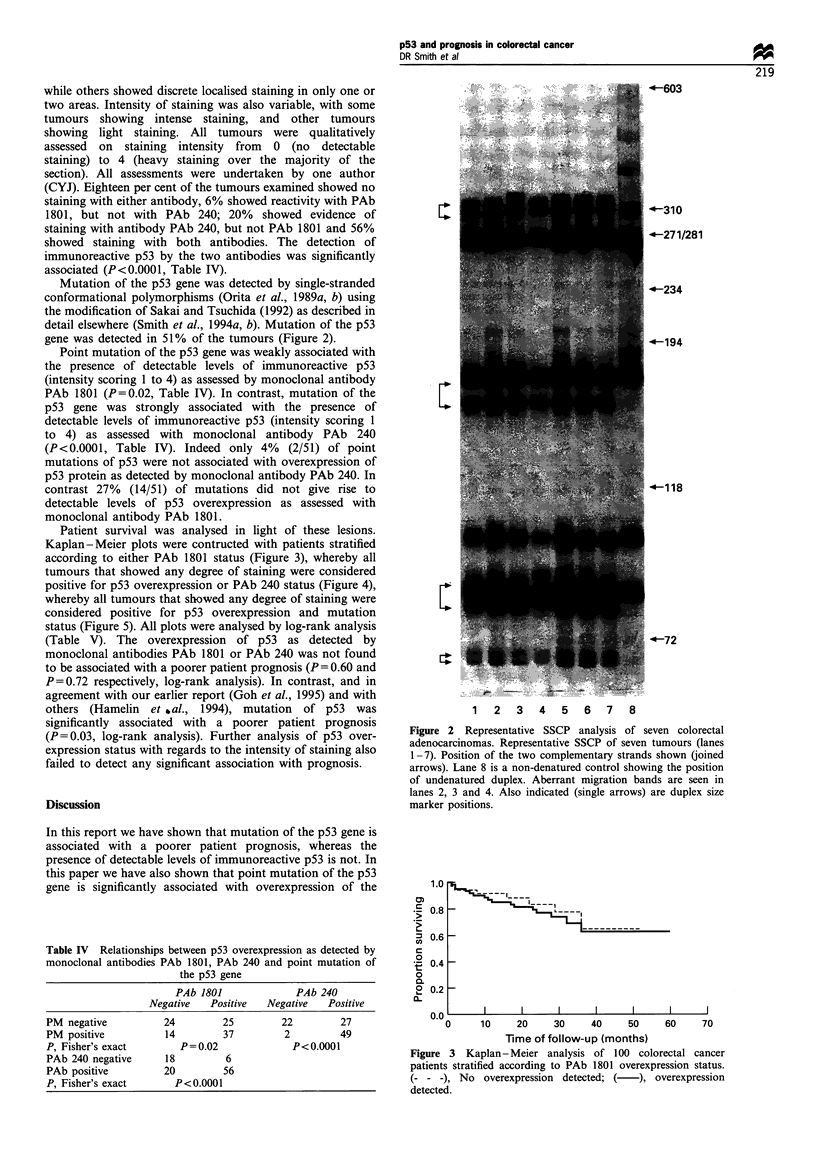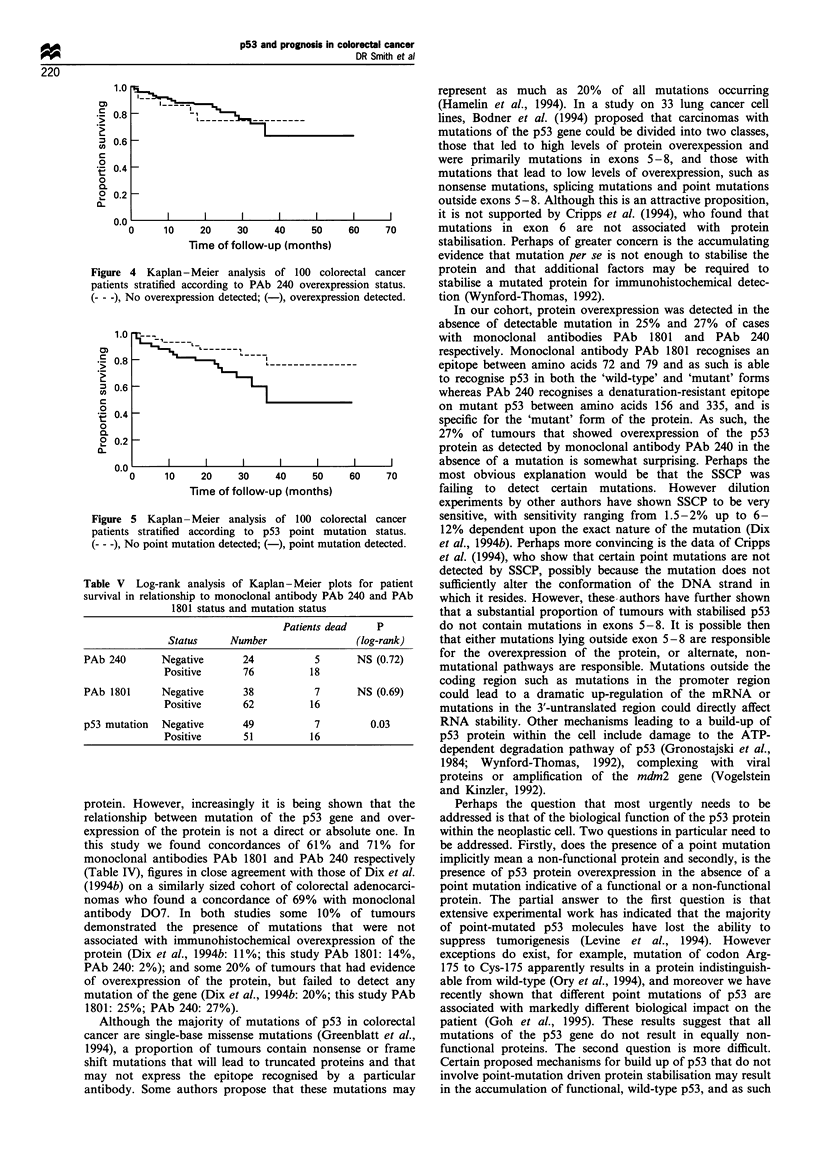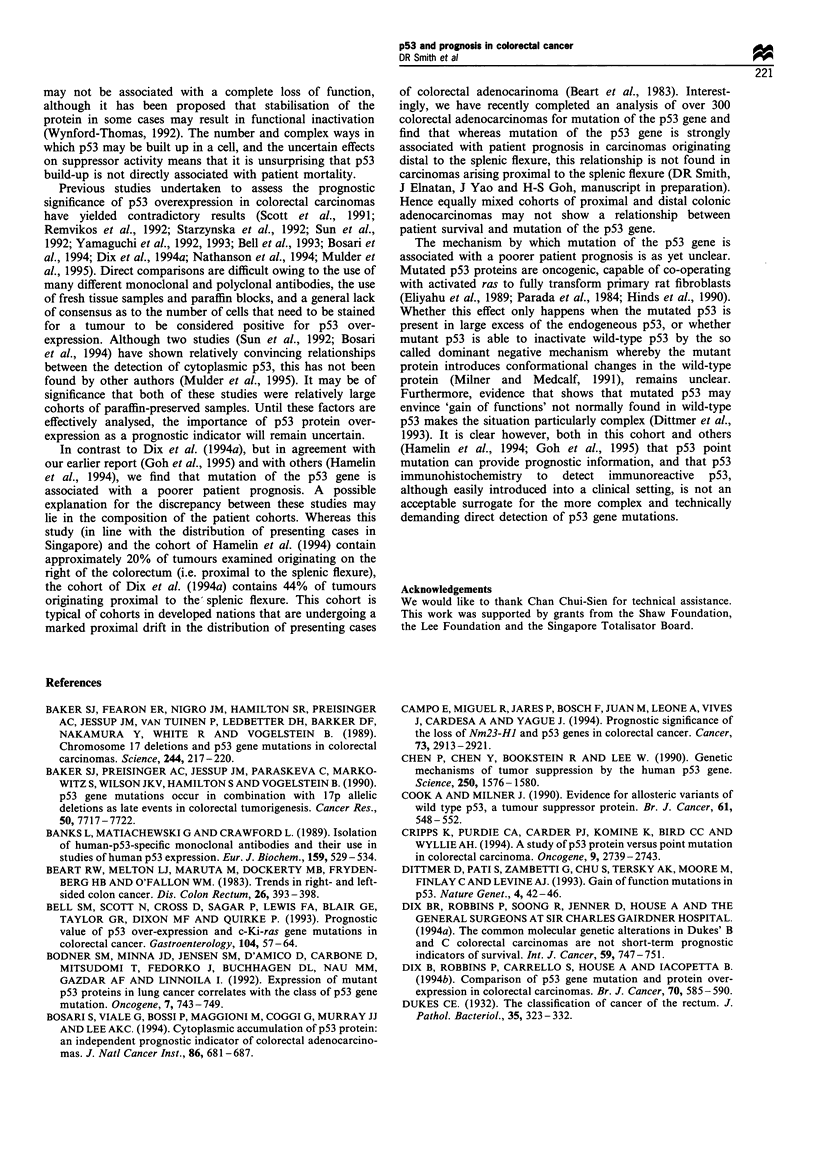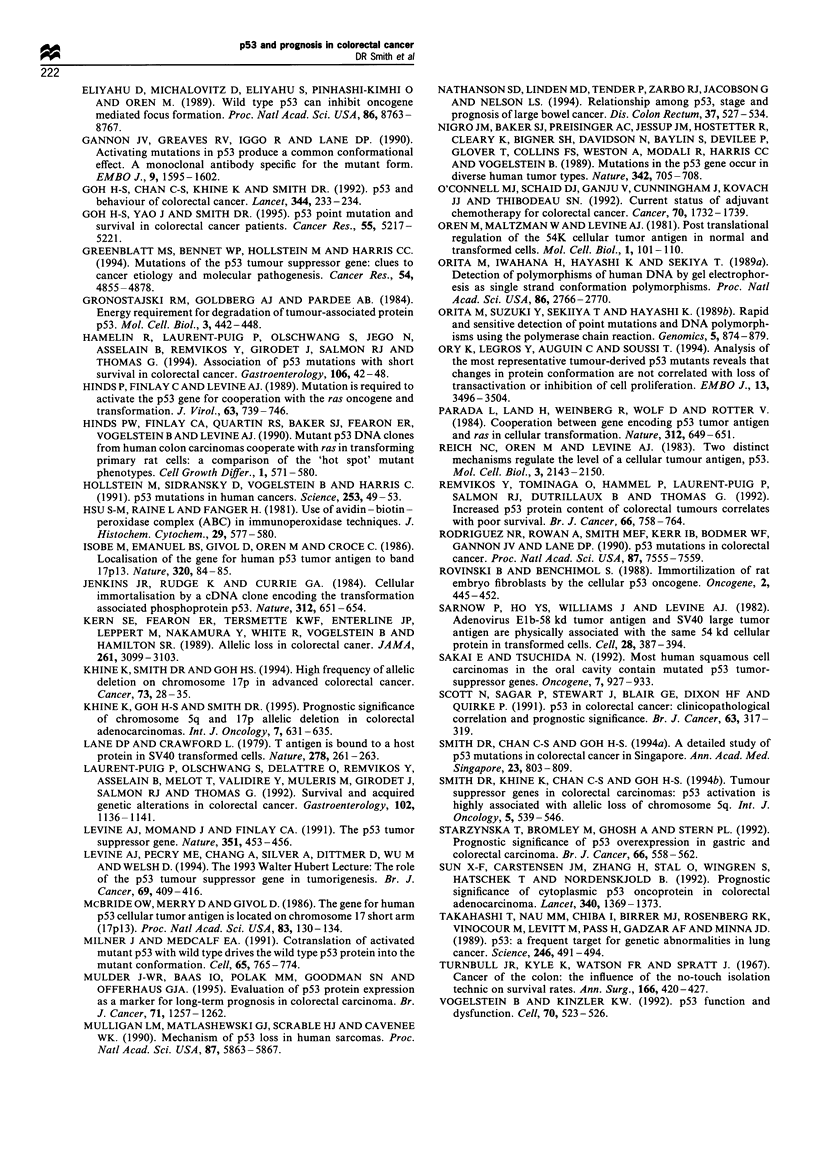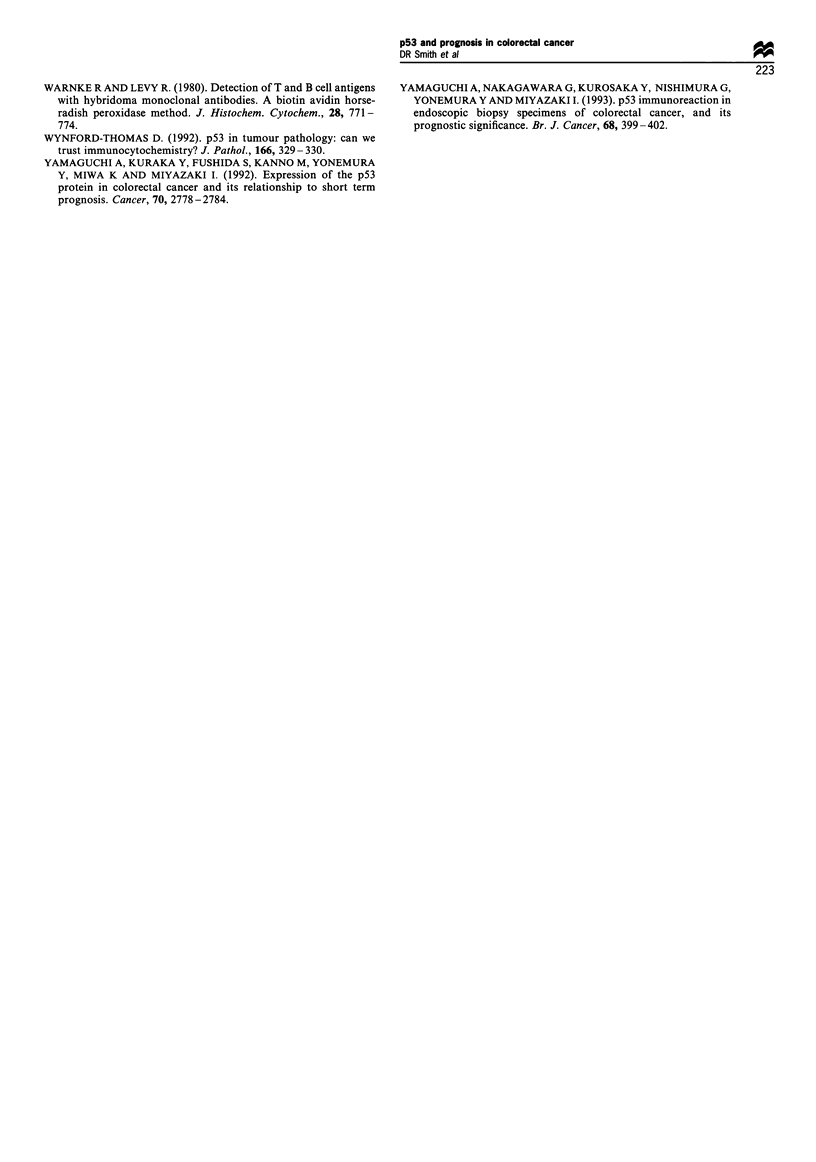# Prognostic significance of p53 overexpression and mutation in colorectal adenocarcinomas.

**DOI:** 10.1038/bjc.1996.340

**Published:** 1996-07

**Authors:** D. R. Smith, C. Y. Ji, H. S. Goh

**Affiliations:** Department of Colorectal Surgery, Singapore General Hospital, Singapore.

## Abstract

**Images:**


British Journal of Cancer (1996) 74, 216-223
© 1996 Stockton Press All rights reserved 0007-0920/96 $12.00

### Prognostic significance of p53 overexpression and mutation in colorectal adenocarcinomas

DR Smith, C-Y Ji and H-S Goh

*Colorectal Cancer Research Laboratory, Department of Colorectal Surgery, Singapore General Hospital, Singapore.*

**Summary** The p53 tumour-suppressor gene is found altered in the majority of colorectal cancers. Lesions include allelic loss, mutation of the gene and overexpression of the p53 protein. All of these lesions have been analysed for prognostic significance, and whereas both mutation and allelic loss have been shown to be reasonably useful markers of prognosis, the utility of overexpression of the p53 protein is more ambiguous. Given that many authors use p53 overexpression as a marker for point mutation this issue is of some importance. We have therefore examined 100 colorectal carcinomas for mutation of the p53 gene, as well as overexpression of the p53 protein. Results show that whereas mutation of the p53 gene is associated with p53 overexpression, the degree of association depends, at least in part, upon the particular antibody used. Moreover, although mutation of the p53 gene does provide prognostic information, overexpresssion of the p53 protein, as detected with two antibodies, does not. These results suggest that immunohistochemistry is not a suitable alternative to direct detection of mutations in assessing prognosis in colorectal cancer patients.

**Keywords:** tumour suppressor; colon cancer

The p53 tumour-suppressor gene is the most commonly altered gene in solid human neoplasia (Hollstein *et al.*, 1991; Levine *et al.*, 1991). The p53 gene was first identified by its ability to complex with SV40 large T antigen (Lane and Crawford, 1979) and the adenovirus type 5 E1B 58KD protein (Sarnow *et al.*, 1982). Localisation of the gene showed that the p53 gene resides at chromosomal location Ch17p13.1 (Isobe *et al.*, 1986; McBride *et al.*, 1986), a site that is frequently found to have undergone allelic deletion in many cancers (Baker *et al.*, 1989; Takahashi *et al.*, 1989; Mulligan *et al.*, 1990). The remaining allele of the p53 gene is commonly found to be mutated (Baker *et al.*, 1989, 1990; Takahashi *et al.*, 1989; Nigro *et al.*, 1989). The wild-type (non-mutated) protein has the ability to reduce or eliminate the tumorigenic potential of a cell line (Chen *et al.*, 1990). The mutated protein has lost this ability, and shows oncogenic activity (Hinds *et al.*, 1989, 1990). Used alone in transformation assays the mutated protein is able to immortalise primary fibroblasts (Jenkins *et al.*, 1984; Rovinski and Benchimol, 1988), and when used in conjunction with activated *ras*, mutated p53 can fully transform primary fibroblasts (Parada *et al.*, 1984; Eliyahu *et al.*, 1989; Hinds *et al.*, 1990).

Wild-type p53 has a short half-life of about 15 min (Oren *et al.*, 1981), and is turned over rapidly by an ATP-dependent degradation pathway (Gronostajski *et al.*, 1984). Mutations within the p53 gene often lead to proteins with a greater stability, with half-lives of up to 20 h in some cases (Oren *et al.*, 1981; Reich *et al.*, 1983). The mutant protein accumulates within the malignant cell and can be readily detected by immunohistochemistry, as opposed to normal cells, in which the protein is essentially undetectable by normal methods (Rodriguez *et al.*, 1990). Mutations of the p53 gene often lead to conformational changes within the p53 protein with the resultant accessibility of epitopes normally unavailable for antibody recognition (Cook and Milner, 1990).

Allelic loss of the p53 gene has been shown to occur in up to 70% of colorectal cancers (Kern *et al.*, 1989; Khine *et al.*, 1994), and is associated with tumour progression, particularly the presence of distant organ metastasis (Khine *et al.*, 1994). In short-term studies, allele loss of p53 does not provide prognostic information (O'Connell *et al.*, 1992; Campo *et al.*, 1994; Khine *et al.*, 1995), but in studies with longer follow-up periods, allelic loss of one copy of the p53 gene is a significant indicator of a poorer patient prognosis (Kern *et al.*, 1989; Laurent-Puig *et al.*, 1992).

Point mutation of the p53 gene occurs in approximately 50% of colorectal carcinomas (Hollstein *et al.*, 1991; Goh *et al.*, 1994), and is also associated with tumour progression (Goh *et al.*, 1994) and a poorer patient prognosis (Hamelin *et al.*, 1994; Goh *et al.*, 1995), although this is not found by some authors (Dix *et al.*, 1994*a*, Table I).

Over-expression of the p53 protein has been reported to occur in 24–72% of colorectal cancers, with a large part of the variation residing in the specificities of the antibodies use in these studies, and to a lesser extent to what the authors define as overexpression (Rodriguez *et al.*, 1990; Scott *et al.*, 1991; Starzynska *et al.*, 1992; Remvikos *et al.*, 1992; Yamaguchi *et al.*, 1992, 1993; Bell *et al.*, 1993; Sun *et al.*, 1992; Bosari *et al.*, 1994; Nathanson *et al.*, 1994; Dix *et al.*, 1994*b*; Mulder *et al.*, 1995). At least 11 studies (see Table II) have examined p53 over-expression with respect to patient survival in colorectal cancer patients (Scott *et al.*, 1991; Remvikos *et al.*, 1992; Starzynska *et al.*, 1992; Sun *et al.*, 1992; Yamaguchi *et al.*, 1992, 1993; Bell *et al.*, 1993; Bosari *et al.*, 1994; Dix *et al.*, 1994*a*; Nathanson *et al.*, 1994; Mulder *et al.*, 1995). Six of these studies found that overexpression of the p53 gene is significantly associated with a poorer patient prognosis in univariate analysis (Remvikos *et al.*, 1992; Starzynska *et al.*, 1992; Yamaguchi *et al.*, 1992, 1993; Bosari *et al.*, 1994; Dix *et al.*, 1994*a*). The remaining five studies do not detect a demonstrable relationship between patient survival and nuclear p53 overexpression (Scott *et al.*, 1991; Sun *et al.*, 1992; Bell *et al.*, 1993; Nathanson *et al.*, 1994; Mulder *et al.*, 1995). Two groups have noted a poorer patient prognosis associated with the detection of immunoreactive p53 in the cytoplasm of colorectal adenocarcinoma cells (Sun *et al.*, 1992; Bosari *et al.*, 1994) although the significance of this remains unclear. In this study we have examined p53 overexpression and patient survival in a cohort in which information on p53 mutation status is also examined.

Correspondence: DR Smith, Brain and Spine Centre, Tan TockSeng Hospital, Moulmein Road, Singapore 308433, Republic of Singapore
Received 21 August 1995; revised 22 January 1996; accepted 1 February 1996

### Materials and methods

### *Patients and tumours*

Samples used in this study were from patients admitted to the Department of Colorectal Surgery at Singapore General Hospital. No initial chemotherapy, radiotherapy or hormonal therapy was given before tumour excision. A portion (approximately 1 g) of the surgically removed tumour was snap frozen in liquid nitrogen at the time of surgery (within 20 min of resection) and stored at −80°C until required. The remainder of the tumour sample was sent for histopathological diagnosis and tumours were staged as Dukes' A–D according to Turnbull's modification of Dukes' original staging (Dukes, 1932; Turnbull *et al.*, 1967). Control mucosa (sited at least 10 cm proximal to the site of the tumour) was also removed and similarly treated. Frozen tumour samples were embedded into OCT freezing media (BDH, Poole, UK), sections taken and stained with haematoxylin–eosin and examined microscopically. Non-tumour regions were then removed and samples processed as below. Patient follow-up (mean 23.4 months, range 1–60 months) was established as the time between surgery and last departmental contact (scheduled follow-up, mail response or telephone contact) or patient death. Death as a result of causes other than cancer were treated as censored events.

### *Immunohistochemistry*

Immunoreactive p53 was detected by the labelled streptavidin–biotin method (Warnke *et al.*, 1980; Hsu *et al.*, 1981). Several contiguous 5 $\mu$m frozen sections were taken from each case. One section was stained with haematoxylin–eosin stain. Sections from immunohistochemistry were allowed to come to room temperature and they dehydrated in acetone for 10 min. Following excessive rinsing in phosphate-buffered saline (PBS) endogenous peroxidases were quenched by incubation in 1.5 hydrogen peroxide, 50% ethanol and 0.5 × PBS for 15 min. Slides were then incubated with blocking reagent for 30 min (Dako LSAB kit, Dako, Carpinteria, CA, USA). Incubation with primary antibody followed rinsing three times with PBS. Incubation at room temperature with a 1:10 dilution of PAb 240 (Oncogene Science, Uniondale, NY, USA) was carried out overnight, and with a 1:40 dilution of PAb 1801 (Oncogene Science) was for 30 min. Following three rinses with PBS slides were incubated with linking antibody (Dako) for 10 min, followed by 10 min with streptavidin-horseradish–peroxidase diluted as recommended by the manufacturer and then incubated for 2 min with the chromagen 3,3′-diaminobenzidine tetrahydrochloride (DAB; Dako). After each incubation samples were rinsed three times in PBS. Samples were then counterstained with haematoxylin for 1 min and nuclei blued under running water. Slides were then dehydrated and mounted.

### *Mutation analysis*

Analysis of colorectal carcinomas was undertaken exactly as described in detail elsewhere (Smith *et al.*, 1994*a*, *b*) and cohort contains samples previously described (Smith *et al.*, 1994*a*, *b*; Goh *et al.*, 1994, 1995). Briefly, a first-strand cDNA copy was made from total RNA using random hexamers. This was used as a template in a polymerase chain reaction to amplify a 644 bp cDNA fragment of p53, which contains the region known to contain 98% of all point mutations of the p53 gene (Hollstein *et al.*, 1991). This fragment was digested with the restriction endonuclease *Msp*I, fragments dephosphorylated with calf intestinal alkaline phosphatase and labelled with $^{32}$P-$\gamma$-ATP. The digestion products were then analysed on a 6% polyacrylamide non-denaturing gel. Samples were analysed by duplicate reverse transcriptase polymerase chain reaction single-stranded conformation polymorphism (RT-PCR-SSCP), and selected cases confirmed by DNA sequencing (Goh *et al.*, 1995). After autoradiography aberrant migration patterns, corresponding to mutations of the p53 gene, can readily be detected.

### *Statistical analysis*

Two by two tables were analysed by Fisher's exact test. Kaplan–Meier survival plots were calculated using the SPSS computer program (SPSS, Chicago, IL, USA) and analysed by log-rank analysis.

**Table I** Summary of studies examing the prognostic significance of p53 point mutation in colorectal cancer

| *Study* | *Cohort size* | *Per cent positive* | *Univariate analysis* |
|---|---|---|---|
| Hamelin *et al.* (1994) | 85 | 52 | $P=0.003$ |
| Goh *et al.* (1995) | 193 | 57 | $P=0.0054$ |
| Dix *et al.* (1994*a*)[a] | 100 | 37 | $P$=NS |
| This study | 100 | 51 | $P=0.03$ |

[a]Dukes' stages B and C only

### Results

A total of 100 colorectal carcinomas were examined for the presence of detectable levels of immunoreactive p53, as well as for mutations of the p53 gene. All tumours were single (non-synchronous, non-metachronous) adenocarcinomas. A clinical summary of patients is shown in Table III. Immunoreactive p53 was detected by the labelled streptavidin–biotin method. At no time was immunoreactive p53

**Table II** Summary of studies examing p53 protein overexpression and patient survival in colorectal cancer

| *Study* | *Tissue type* | *Cohort size* | *Antibody* | *Per cent positive* | *Univariate analysis* |
|---|---|---|---|---|---|
| Bell *et al.* (1993) | Fresh, frozen | 100[b] | PAB 421 | 45 | $P$=NS |
| Bosari *et al.* (1994)[a] | Parrafin block | 206 | PAb 1801 | 46 | $P=0.019$ |
| Dix *et al.* (1994*a*) | Fresh, frozen | 100 | DO7 | 46 | $P=0.039$ |
| Mulder *et al.* (1995) | Paraffin block | 109 | DO7 | 72 | $P$=NS |
| Nathanson *et al.* (1994) | Paraffin block | 84 | PAb 1801 | 62 | $P$=NS |
| Remvikos *et al.* (1992) | Fresh, frozen[b] | 78 | PAb 240 | 67 | $P=0.03$ |
| Scott *et al.* (1991) | Fresh, frozen | 52 | PAb 421 | 42 | $P$=NS |
| Starzynska *et al.* (1992) | Paraffin block | 107 | CM1 | 46 | $P=0.001$ |
| Sun *et al.* (1992)[a] | Paraffin block | 293 | CM1 | 49 | $P$=NS |
| Yamaguchi *et al.* (1992) | Paraffin block | 100 | 1801 | 61 | $P<0.05$ |
| Yamaguchi *et al.* (1993) | Parrafin block | 203 | 1801 | 59.6 | $P<0.05$ |
| This study | Fresh, frozen | 100 | 240 | 76 | $P$=NS |
| | | | 1801 | 62 | $P$=NS |

[a]Cytoplasmic staining associated with poorer patient prognosis. [b]p53 content analysed by flow cytometry and ELISA.

detected in histologically normal control mucosa. Antibodies PAb 1801 (Banks *et al.*, 1989) and PAb 240 (Gannon *et al.*, 1990) were used to analyse each specimen, as evidence has shown that not all antibodies have comparable reactivity (Cook and Milner, 1990). Antibody PAb 1801 recognises an epitope of the p53 protein between amino acids 72 and 79, whereas PAb 240 recognises a denaturing-resistant epitope of p53 between amino acids 156 and 335.

Immunoreactive p53 was detected in 62% of cases with monoclonal antibody PAb 1801, and in 76% of cases with monoclonal antibody PAb 240 (Figure 1). As has been reported by other workers staining was predominantly nuclear (Rodriguez *et al.*, 1990; Scott *et al.*, 1991; Remvikos *et al.*, 1992; Starzynska *et al.*, 1992; Sun *et al.*, 1992; Yamaguchi *et al.*, 1992; Bell *et al.*, 1993; Bosari *et al.*, 1994), although some cases of cytoplasmic staining were noted. The distribution of staining was variable with some tumours showing extensive staining over almost the whole section

**Table III** Clinical summary of patients in relation to p53 overexpression and mutation of the gene

| | | *PAb 240* | | *PAb 1801* | | *Mutation* | |
|---|---|---|---|---|---|---|---|
| | *Total* | *Negative* | *Positive* | *Negative* | *Positive* | *Negative* | *Positive* |
| Number of patients | 100 | 24 | 76 | 38 | 62 | 49 | 51 |
| Sex | | | | | | | |
| Male | 53 | 10 | 43 | 18 | 35 | 26 | 27 |
| Female | 47 | 14 | 33 | 20 | 27 | 23 | 24 |
| Age | | | | | | | |
| Mean | 64.0 | 66.5 | 63.2 | 64.8 | 63.4 | 63.8 | 64.1 |
| Range | 28-87 | 28-82 | 33-87 | 28-87 | 33-84 | 28-82 | 33-87 |
| Location | | | | | | | |
| Proximal | 22 | 7 | 15 | 8 | 14 | 15 | 7 |
| Distal | 78 | 17 | 61 | 30 | 48 | 34 | 44 |
| Tumour stage | | | | | | | |
| Dukes' A | 22 | 5 | 17 | 8 | 14 | 13 | 9 |
| Dukes' B | 23 | 6 | 17 | 13 | 9 | 12 | 10 |
| Dukes' C | 32 | 5 | 27 | 10 | 22 | 12 | 20 |
| Dukes' D | 24 | 8 | 16 | 7 | 17 | 12 | 12 |
| Status | | | | | | | |
| Alive | 77 | 19 | 58 | 31 | 46 | 42 | 35 |
| Dead | 23 | 5 | 18 | 7 | 16 | 7 | 16 |

**Figure 1** Overexpression of p53 in colorectal adenocarcinoma. Figure shows immunostaining with monoclonal antibodies PAb 240 (**a**, **c**) and PAb 1801 (**b**). Also shown is a negative control (**d**, minus primary antibody). Immunoreactive p53 stains brown. Original magnification × 100 (**a**, **b**) × 400 (**c**, **d**).

while others showed discrete localised staining in only one or two areas. Intensity of staining was also variable, with some tumours showing intense staining, and other tumours showing light staining. All tumours were qualitatively assessed on staining intensity from 0 (no detectable staining) to 4 (heavy staining over the majority of the section). All assessments were undertaken by one author (CYJ). Eighteen per cent of the tumours examined showed no staining with either antibody, 6% showed reactivity with PAb 1801, but not with PAb 240; 20% showed evidence of staining with antibody PAb 240, but not PAb 1801 and 56% showed staining with both antibodies. The detection of immunoreactive p53 by the two antibodies was significantly associated ($P$<0.0001, Table IV).

Mutation of the p53 gene was detected by single-stranded conformational polymorphisms (Orita *et al.*, 1989*a*, *b*) using the modification of Sakai and Tsuchida (1992) as described in detail elsewhere (Smith *et al.*, 1994*a*, *b*). Mutation of the p53 gene was detected in 51% of the tumours (Figure 2).

Point mutation of the p53 gene was weakly associated with the presence of detectable levels of immunoreactive p53 (intensity scoring 1 to 4) as assessed by monoclonal antibody PAb 1801 ($P$=0.02, Table IV). In contrast, mutation of the p53 gene was strongly associated with the presence of detectable levels of immunoreactive p53 (intensity scoring 1 to 4) as assessed with monoclonal antibody PAb 240 ($P$<0.0001, Table IV). Indeed only 4% (2/51) of point mutations of p53 were not associated with overexpression of p53 protein as detected by monoclonal antibody PAb 240. In contrast 27% (14/51) of mutations did not give rise to detectable levels of p53 overexpression as assessed with monoclonal antibody PAb 1801.

Patient survival was analysed in light of these lesions. Kaplan–Meier plots were contructed with patients stratified according to either PAb 1801 status (Figure 3), whereby all tumours that showed any degree of staining were considered positive for p53 overexpression or PAb 240 status (Figure 4), whereby all tumours that showed any degree of staining were considered positive for p53 overexpression and mutation status (Figure 5). All plots were analysed by log-rank analysis (Table V). The overexpression of p53 as detected by monoclonal antibodies PAb 1801 or PAb 240 was not found to be associated with a poorer patient prognosis ($P$=0.60 and $P$=0.72 respectively, log-rank analysis). In contrast, and in agreement with our earlier report (Goh *et al.*, 1995) and with others (Hamelin *et al.*, 1994), mutation of p53 was significantly associated with a poorer patient prognosis ($P$=0.03, log-rank analysis). Further analysis of p53 overexpression status with regards to the intensity of staining also failed to detect any significant association with prognosis.

### Discussion

In this report we have shown that mutation of the p53 gene is associated with a poorer patient prognosis, whereas the presence of detectable levels of immunoreactive p53 is not. In this paper we have also shown that point mutation of the p53 gene is significantly associated with overexpression of the

**Table IV** Relationships between p53 overexpression as detected by monoclonal antibodies PAb 1801, PAb 240 and point mutation of the p53 gene

| | *PAb 1801* | | *PAb 240* | |
|---|---|---|---|---|
| | *Negative* | *Positive* | *Negative* | *Positive* |
| PM negative | 24 | 25 | 22 | 27 |
| PM positive | 14 | 37 | 2 | 49 |
| $P$, Fisher's exact | $P$=0.02 | | $P$<0.0001 | |
| PAb 240 negative | 18 | 6 | | |
| PAb positive | 20 | 56 | | |
| $P$, Fisher's exact | $P$<0.0001 | | | |

**Figure 2** Representative SSCP analysis of seven colorectal adenocarcinomas. Representative SSCP of seven tumours (lanes 1–7). Position of the two complementary strands shown (joined arrows). Lane 8 is a non-denatured control showing the position of undenatured duplex. Aberrant migration bands are seen in lanes 2, 3 and 4. Also indicated (single arrows) are duplex size marker positions.

**Figure 3** Kaplan–Meier analysis of 100 colorectal cancer patients stratified according to PAb 1801 overexpression status. (- - -), No overexpression detected; (——), overexpression detected.

**Figure 4** Kaplan–Meier analysis of 100 colorectal cancer patients stratified according to PAb 240 overexpression status. (- - -), No overexpression detected; (—), overexpression detected.

**Figure 5** Kaplan–Meier analysis of 100 colorectal cancer patients stratified according to p53 point mutation status. (- - -), No point mutation detected; (—), point mutation detected.

**Table V** Log-rank analysis of Kaplan–Meier plots for patient survival in relationship to monoclonal antibody PAb 240 and PAb 1801 status and mutation status

| | *Status* | *Number* | *Patients dead* | P (*log-rank*) |
|---|---|---|---|---|
| PAb 240 | Negative | 24 | 5 | NS (0.72) |
| | Positive | 76 | 18 | |
| PAb 1801 | Negative | 38 | 7 | NS (0.69) |
| | Positive | 62 | 16 | |
| p53 mutation | Negative | 49 | 7 | 0.03 |
| | Positive | 51 | 16 | |

protein. However, increasingly it is being shown that the relationship between mutation of the p53 gene and overexpression of the protein is not a direct or absolute one. In this study we found concordances of 61% and 71% for monoclonal antibodies PAb 1801 and PAb 240 respectively (Table IV), figures in close agreement with those of Dix *et al.* (1994*b*) on a similarly sized cohort of colorectal adenocarcinomas who found a concordance of 69% with monoclonal antibody DO7. In both studies some 10% of tumours demonstrated the presence of mutations that were not associated with immunohistochemical overexpression of the protein (Dix *et al.*, 1994*b*: 11%; this study PAb 1801: 14%, PAb 240: 2%); and some 20% of tumours that had evidence of overexpression of the protein, but failed to detect any mutation of the gene (Dix *et al.*, 1994*b*: 20%; this study PAb 1801: 25%; PAb 240: 27%).

Although the majority of mutations of p53 in colorectal cancer are single-base missense mutations (Greenblatt *et al.*, 1994), a proportion of tumours contain nonsense or frame shift mutations that will lead to truncated proteins and that may not express the epitope recognised by a particular antibody. Some authors propose that these mutations may represent as much as 20% of all mutations occurring (Hamelin *et al.*, 1994). In a study on 33 lung cancer cell lines, Bodner *et al.* (1994) proposed that carcinomas with mutations of the p53 gene could be divided into two classes, those that led to high levels of protein overexpression and were primarily mutations in exons 5–8, and those with mutations that lead to low levels of overexpression, such as nonsense mutations, splicing mutations and point mutations outside exons 5–8. Although this is an attractive proposition, it is not supported by Cripps *et al.* (1994), who found that mutations in exon 6 are not associated with protein stabilisation. Perhaps of greater concern is the accumulating evidence that mutation *per se* is not enough to stabilise the protein and that additional factors may be required to stabilise a mutated protein for immunohistochemical detection (Wynford-Thomas, 1992).

In our cohort, protein overexpression was detected in the absence of detectable mutation in 25% and 27% of cases with monoclonal antibodies PAb 1801 and PAb 240 respectively. Monoclonal antibody PAb 1801 recognises an epitope between amino acids 72 and 79 and as such is able to recognise p53 in both the 'wild-type' and 'mutant' forms whereas PAb 240 recognises a denaturation-resistant epitope on mutant p53 between amino acids 156 and 335, and is specific for the 'mutant' form of the protein. As such, the 27% of tumours that showed overexpression of the p53 protein as detected by monoclonal antibody PAb 240 in the absence of a mutation is somewhat surprising. Perhaps the most obvious explanation would be that the SSCP was failing to detect certain mutations. However dilution experiments by other authors have shown SSCP to be very sensitive, with sensitivity ranging from 1.5–2% up to 6–12% dependent upon the exact nature of the mutation (Dix *et al.*, 1994*b*). Perhaps more convincing is the data of Cripps *et al.* (1994), who show that certain point mutations are not detected by SSCP, possibly because the mutation does not sufficiently alter the conformation of the DNA strand in which it resides. However, these authors have further shown that a substantial proportion of tumours with stabilised p53 do not contain mutations in exons 5–8. It is possible then that either mutations lying outside exon 5–8 are responsible for the overexpression of the protein, or alternate, non-mutational pathways are responsible. Mutations outside the coding region such as mutations in the promoter region could lead to a dramatic up-regulation of the mRNA or mutations in the 3′-untranslated region could directly affect RNA stability. Other mechanisms leading to a build-up of p53 protein within the cell include damage to the ATP-dependent degradation pathway of p53 (Gronostajski *et al.*, 1984; Wynford-Thomas, 1992), complexing with viral proteins or amplification of the *mdm*2 gene (Vogelstein and Kinzler, 1992).

Perhaps the question that most urgently needs to be addressed is that of the biological function of the p53 protein within the neoplastic cell. Two questions in particular need to be addressed. Firstly, does the presence of a point mutation implicitly mean a non-functional protein and secondly, is the presence of p53 protein overexpression in the absence of a point mutation indicative of a functional or a non-functional protein. The partial answer to the first question is that extensive experimental work has indicated that the majority of point-mutated p53 molecules have lost the ability to suppress tumorigenesis (Levine *et al.*, 1994). However exceptions do exist, for example, mutation of codon Arg-175 to Cys-175 apparently results in a protein indistinguishable from wild-type (Ory *et al.*, 1994), and moreover we have recently shown that different point mutations of p53 are associated with markedly different biological impact on the patient (Goh *et al.*, 1995). These results suggest that all mutations of the p53 gene do not result in equally non-functional proteins. The second question is more difficult. Certain proposed mechanisms for build up of p53 that do not involve point-mutation driven protein stabilisation may result in the accumulation of functional, wild-type p53, and as such may not be associated with a complete loss of function, although it has been proposed that stabilisation of the protein in some cases may result in functional inactivation (Wynford-Thomas, 1992). The number and complex ways in which p53 may be built up in a cell, and the uncertain effects on suppressor activity means that it is unsurprising that p53 build-up is not directly associated with patient mortality.

Previous studies undertaken to assess the prognostic significance of p53 overexpression in colorectal carcinomas have yielded contradictory results (Scott *et al.*, 1991; Remvikos *et al.*, 1992; Starzynska *et al.*, 1992; Sun *et al.*, 1992; Yamaguchi *et al.*, 1992, 1993; Bell *et al.*, 1993; Bosari *et al.*, 1994; Dix *et al.*, 1994*a*; Nathanson *et al.*, 1994; Mulder *et al.*, 1995). Direct comparisons are difficult owing to the use of many different monoclonal and polyclonal antibodies, the use of fresh tissue samples and paraffin blocks, and a general lack of consensus as to the number of cells that need to be stained for a tumour to be considered positive for p53 overexpression. Although two studies (Sun *et al.*, 1992; Bosari *et al.*, 1994) have shown relatively convincing relationships between the detection of cytoplasmic p53, this has not been found by other authors (Mulder *et al.*, 1995). It may be of significance that both of these studies were relatively large cohorts of paraffin-preserved samples. Until these factors are effectively analysed, the importance of p53 protein overexpression as a prognostic indicator will remain uncertain.

In contrast to Dix *et al.* (1994*a*), but in agreement with our earlier report (Goh *et al.*, 1995) and with others (Hamelin *et al.*, 1994), we find that mutation of the p53 gene is associated with a poorer patient prognosis. A possible explanation for the discrepancy between these studies may lie in the composition of the patient cohorts. Whereas this study (in line with the distribution of presenting cases in Singapore) and the cohort of Hamelin *et al.* (1994) contain approximately 20% of tumours examined originating on the right of the colorectum (i.e. proximal to the splenic flexure), the cohort of Dix *et al.* (1994*a*) contains 44% of tumours originating proximal to the splenic flexure. This cohort is typical of cohorts in developed nations that are undergoing a marked proximal drift in the distribution of presenting cases of colorectal adenocarinoma (Beart *et al.*, 1983). Interestingly, we have recently completed an analysis of over 300 colorectal adenocarcinomas for mutation of the p53 gene and find that whereas mutation of the p53 gene is strongly associated with patient prognosis in carcinomas originating distal to the splenic flexure, this relationship is not found in carcinomas arising proximal to the splenic flexure (DR Smith, J Elnatan, J Yao and H-S Goh, manuscript in preparation). Hence equally mixed cohorts of proximal and distal colonic adenocarcinomas may not show a relationship between patient survival and mutation of the p53 gene.

The mechanism by which mutation of the p53 gene is associated with a poorer patient prognosis is as yet unclear. Mutated p53 proteins are oncogenic, capable of co-operating with activated *ras* to fully transform primary rat fibroblasts (Eliyahu *et al.*, 1989; Parada *et al.*, 1984; Hinds *et al.*, 1990). Whether this effect only happens when the mutated p53 is present in large excess of the endogeneous p53, or whether mutant p53 is able to inactivate wild-type p53 by the so called dominant negative mechanism whereby the mutant protein introduces conformational changes in the wild-type protein (Milner and Medcalf, 1991), remains unclear. Furthermore, evidence that shows that mutated p53 may envince 'gain of functions' not normally found in wild-type p53 makes the situation particularly complex (Dittmer *et al.*, 1993). It is clear however, both in this cohort and others (Hamelin *et al.*, 1994; Goh *et al.*, 1995) that p53 point mutation can provide prognostic information, and that p53 immunohistochemistry to detect immunoreactive p53, although easily introduced into a clinical setting, is not an acceptable surrogate for the more complex and technically demanding direct detection of p53 gene mutations.

**Acknowledgements**
We would like to thank Chan Chui-Sien for technical assistance. This work was supported by grants from the Shaw Foundation, the Lee Foundation and the Singapore Totalisator Board.

### References

BAKER SJ, FEARON ER, NIGRO JM, HAMILTON SR, PREISINGER AC, JESSUP JM, VAN TUINEN P, LEDBETTER DH, BARKER DF, NAKAMURA Y, WHITE R AND VOGELSTEIN B. (1989). Chromosome 17 deletions and p53 gene mutations in colorectal carcinomas. *Science*, **244**, 217-220.

BAKER SJ, PREISINGER AC, JESSUP JM, PARASKEVA C, MARKOWITZ S, WILSON JKV, HAMILTON S AND VOGELSTEIN B. (1990). p53 gene mutations occur in combination with 17p allelic deletions as late events in colorectal tumorigenesis. *Cancer Res.*, **50**, 7717-7722.

BANKS L, MATIACHEWSKI G AND CRAWFORD L. (1989). Isolation of human-p53-specific monoclonal antibodies and their use in studies of human p53 expression. *Eur. J. Biochem.*, **159**, 529-534.

BEART RW, MELTON LJ, MARUTA M, DOCKERTY MB, FRYDENBERG HB AND O'FALLON WM. (1983). Trends in right- and left-sided colon cancer. *Dis. Colon Rectum*, **26**, 393-398.

BELL SM, SCOTT N, CROSS D, SAGAR P, LEWIS FA, BLAIR GE, TAYLOR GR, DIXON MF AND QUIRKE P. (1993). Prognostic value of p53 over-expression and c-Ki-*ras* gene mutations in colorectal cancer. *Gastroenterology*, **104**, 57-64.

BODNER SM, MINNA JD, JENSEN SM, D'AMICO D, CARBONE D, MITSUDOMI T, FEDORKO J, BUCHHAGEN DL, NAU MM, GAZDAR AF AND LINNOILA I. (1992). Expression of mutant p53 proteins in lung cancer correlates with the class of p53 gene mutation. *Oncogene*, **7**, 743-749.

BOSARI S, VIALE G, BOSSI P, MAGGIONI M, COGGI G, MURRAY JJ AND LEE AKC. (1994). Cytoplasmic accumulation of p53 protein: an independent prognostic indicator of colorectal adenocarcinomas. *J. Natl Cancer Inst.*, **86**, 681-687.

CAMPO E, MIGUEL R, JARES P, BOSCH F, JUAN M, LEONE A, VIVES J, CARDESA A AND YAGUE J. (1994). Prognostic significance of the loss of *Nm23-H1* and p53 genes in colorectal cancer. *Cancer*, **73**, 2913-2921.

CHEN P, CHEN Y, BOOKSTEIN R AND LEE W. (1990). Genetic mechanisms of tumor suppression by the human p53 gene. *Science*, **250**, 1576-1580.

COOK A AND MILNER J. (1990). Evidence for allosteric variants of wild type p53, a tumour suppressor protein. *Br. J. Cancer*, **61**, 548-552.

CRIPPS K, PURDIE CA, CARDER PJ, KOMINE K, BIRD CC AND WYLLIE AH. (1994). A study of p53 protein versus point mutation in colorectal carcinoma. *Oncogene*, **9**, 2739-2743.

DITTMER D, PATI S, ZAMBETTI G, CHU S, TERSKY AK, MOORE M, FINLAY C AND LEVINE AJ. (1993). Gain of function mutations in p53. *Nature Genet.*, **4**, 42-46.

DIX BR, ROBBINS P, SOONG R, JENNER D, HOUSE A AND THE GENERAL SURGEONS AT SIR CHARLES GAIRDNER HOSPITAL. (1994*a*). The common molecular genetic alterations in Dukes' B and C colorectal carcinomas are not short-term prognostic indicators of survival. *Int. J. Cancer*, **59**, 747-751.

DIX B, ROBBINS P, CARRELLO S, HOUSE A AND IACOPETTA B. (1994*b*). Comparison of p53 gene mutation and protein overexpression in colorectal carcinomas. *Br. J. Cancer*, **70**, 585-590.

DUKES CE. (1932). The classification of cancer of the rectum. *J. Pathol. Bacteriol.*, **35**, 323-332.

ELIYAHU D, MICHALOVITZ D, ELIYAHU S, PINHASHI-KIMHI O AND OREN M. (1989). Wild type p53 can inhibit oncogene mediated focus formation. *Proc. Natl Acad. Sci. USA*, **86**, 8763–8767.

GANNON JV, GREAVES RV, IGGO R AND LANE DP. (1990). Activating mutations in p53 produce a common conformational effect. A monoclonal antibody specific for the mutant form. *EMBO J.*, **9**, 1595–1602.

GOH H-S, CHAN C-S, KHINE K AND SMITH DR. (1992). p53 and behaviour of colorectal cancer. *Lancet*, **344**, 233–234.

GOH H-S, YAO J AND SMITH DR. (1995). p53 point mutation and survival in colorectal cancer patients. *Cancer Res.*, **55**, 5217–5221.

GREENBLATT MS, BENNET WP, HOLLSTEIN M AND HARRIS CC. (1994). Mutations of the p53 tumour suppressor gene: clues to cancer etiology and molecular pathogenesis. *Cancer Res.*, **54**, 4855–4878.

GRONOSTAJSKI RM, GOLDBERG AJ AND PARDEE AB. (1984). Energy requirement for degradation of tumour-associated protein p53. *Mol. Cell. Biol.*, **3**, 442–448.

HAMELIN R, LAURENT-PUIG P, OLSCHWANG S, JEGO N, ASSELAIN B, REMVIKOS Y, GIRODET J, SALMON RJ AND THOMAS G. (1994). Association of p53 mutations with short survival in colorectal cancer. *Gastroenterology*, **106**, 42–48.

HINDS P, FINLAY C AND LEVINE AJ. (1989). Mutation is required to activate the p53 gene for cooperation with the *ras* oncogene and transformation. *J. Virol.*, **63**, 739–746.

HINDS PW, FINLAY CA, QUARTIN RS, BAKER SJ, FEARON ER, VOGELSTEIN B AND LEVINE AJ. (1990). Mutant p53 DNA clones from human colon carcinomas cooperate with *ras* in transforming primary rat cells: a comparison of the 'hot spot' mutant phenotypes. *Cell Growth Differ.*, **1**, 571–580.

HOLLSTEIN M, SIDRANSKY D, VOGELSTEIN B AND HARRIS C. (1991). p53 mutations in human cancers. *Science*, **253**, 49–53.

HSU S-M, RAINE L AND FANGER H. (1981). Use of avidin–biotin–peroxidase complex (ABC) in immunoperoxidase techniques. *J. Histochem. Cytochem.*, **29**, 577–580.

ISOBE M, EMANUEL BS, GIVOL D, OREN M AND CROCE C. (1986). Localisation of the gene for human p53 tumor antigen to band 17p13. *Nature*, **320**, 84–85.

JENKINS JR, RUDGE K AND CURRIE GA. (1984). Cellular immortalisation by a cDNA clone encoding the transformation associated phosphoprotein p53. *Nature*, **312**, 651–654.

KERN SE, FEARON ER, TERSMETTE KWF, ENTERLINE JP, LEPPERT M, NAKAMURA Y, WHITE R, VOGELSTEIN B AND HAMILTON SR. (1989). Allelic loss in colorectal caner. *JAMA*, **261**, 3099–3103.

KHINE K, SMITH DR AND GOH HS. (1994). High frequency of allelic deletion on chromosome 17p in advanced colorectal cancer. *Cancer*, **73**, 28–35.

KHINE K, GOH H-S AND SMITH DR. (1995). Prognostic significance of chromosome 5q and 17p allelic deletion in colorectal adenocarcinomas. *Int. J. Oncology*, **7**, 631–635.

LANE DP AND CRAWFORD L. (1979). T antigen is bound to a host protein in SV40 transformed cells. *Nature*, **278**, 261–263.

LAURENT-PUIG P, OLSCHWANG S, DELATTRE O, REMVIKOS Y, ASSELAIN B, MELOT T, VALIDIRE Y, MULERIS M, GIRODET J, SALMON RJ AND THOMAS G. (1992). Survival and acquired genetic alterations in colorectal cancer. *Gastroenterology*, **102**, 1136–1141.

LEVINE AJ, MOMAND J AND FINLAY CA. (1991). The p53 tumor suppressor gene. *Nature*, **351**, 453–456.

LEVINE AJ, PECRY ME, CHANG A, SILVER A, DITTMER D, WU M AND WELSH D. (1994). The 1993 Walter Hubert Lecture: The role of the p53 tumour suppressor gene in tumorigenesis. *Br. J. Cancer*, **69**, 409–416.

McBRIDE OW, MERRY D AND GIVOL D. (1986). The gene for human p53 cellular tumor antigen is located on chromosome 17 short arm (17p13). *Proc. Natl Acad. Sci. USA*, **83**, 130–134.

MILNER J AND MEDCALF EA. (1991). Cotranslation of activated mutant p53 with wild type drives the wild type p53 protein into the mutant conformation. *Cell*, **65**, 765–774.

MULDER J-WR, BAAS IO, POLAK MM, GOODMAN SN AND OFFERHAUS GJA. (1995). Evaluation of p53 protein expression as a marker for long-term prognosis in colorectal carcinoma. *Br. J. Cancer*, **71**, 1257–1262.

MULLIGAN LM, MATLASHEWSKI GJ, SCRABLE HJ AND CAVENEE WK. (1990). Mechanism of p53 loss in human sarcomas. *Proc. Natl Acad. Sci. USA*, **87**, 5863–5867.

NATHANSON SD, LINDEN MD, TENDER P, ZARBO RJ, JACOBSON G AND NELSON LS. (1994). Relationship among p53, stage and prognosis of large bowel cancer. *Dis. Colon Rectum*, **37**, 527–534.

NIGRO JM, BAKER SJ, PREISINGER AC, JESSUP JM, HOSTETTER R, CLEARY K, BIGNER SH, DAVIDSON N, BAYLIN S, DEVILEE P, GLOVER T, COLLINS FS, WESTON A, MODALI R, HARRIS CC AND VOGELSTEIN B. (1989). Mutations in the p53 gene occur in diverse human tumor types. *Nature*, **342**, 705–708.

O'CONNELL MJ, SCHAID DJ, GANJU V, CUNNINGHAM J, KOVACH JJ AND THIBODEAU SN. (1992). Current status of adjuvant chemotherapy for colorectal cancer. *Cancer*, **70**, 1732–1739.

OREN M, MALTZMAN W AND LEVINE AJ. (1981). Post translational regulation of the 54K cellular tumor antigen in normal and transformed cells. *Mol. Cell. Biol.*, **1**, 101–110.

ORITA M, IWAHANA H, HAYASHI K AND SEKIYA T. (1989*a*). Detection of polymorphisms of human DNA by gel electrophoresis as single strand conformation polymorphisms. *Proc. Natl Acad. Sci. USA*, **86**, 2766–2770.

ORITA M, SUZUKI Y, SEKIIYA T AND HAYASHI K. (1989*b*). Rapid and sensitive detection of point mutations and DNA polymorphisms using the polymerase chain reaction. *Genomics*, **5**, 874–879.

ORY K, LEGROS Y, AUGUIN C AND SOUSSI T. (1994). Analysis of the most representative tumour-derived p53 mutants reveals that changes in protein conformation are not correlated with loss of transactivation or inhibition of cell proliferation. *EMBO J.*, **13**, 3496–3504.

PARADA L, LAND H, WEINBERG R, WOLF D AND ROTTER V. (1984). Cooperation between gene encoding p53 tumor antigen and *ras* in cellular transformation. *Nature*, **312**, 649–651.

REICH NC, OREN M AND LEVINE AJ. (1983). Two distinct mechanisms regulate the level of a cellular tumour antigen, p53. *Mol. Cell. Biol.*, **3**, 2143–2150.

REMVIKOS Y, TOMINAGA O, HAMMEL P, LAURENT-PUIG P, SALMON RJ, DUTRILLAUX B AND THOMAS G. (1992). Increased p53 protein content of colorectal tumours correlates with poor survival. *Br. J. Cancer*, **66**, 758–764.

RODRIGUEZ NR, ROWAN A, SMITH MEF, KERR IB, BODMER WF, GANNON JV AND LANE DP. (1990). p53 mutations in colorectal cancer. *Proc. Natl Acad. Sci. USA*, **87**, 7555–7559.

ROVINSKI B AND BENCHIMOL S. (1988). Immortilization of rat embryo fibroblasts by the cellular p53 oncogene. *Oncogene*, **2**, 445–452.

SARNOW P, HO YS, WILLIAMS J AND LEVINE AJ. (1982). Adenovirus E1b-58 kd tumor antigen and SV40 large tumor antigen are physically associated with the same 54 kd cellular protein in transformed cells. *Cell*, **28**, 387–394.

SAKAI E AND TSUCHIDA N. (1992). Most human squamous cell carcinomas in the oral cavity contain mutated p53 tumor-suppressor genes. *Oncogene*, **7**, 927–933.

SCOTT N, SAGAR P, STEWART J, BLAIR GE, DIXON HF AND QUIRKE P. (1991). p53 in colorectal cancer: clinicopathological correlation and prognostic significance. *Br. J. Cancer*, **63**, 317–319.

SMITH DR, CHAN C-S AND GOH H-S. (1994*a*). A detailed study of p53 mutations in colorectal cancer in Singapore. *Ann. Acad. Med. Singapore*, **23**, 803–809.

SMITH DR, KHINE K, CHAN C-S AND GOH H-S. (1994*b*). Tumour suppressor genes in colorectal carcinomas: p53 activation is highly associated with allelic loss of chromosome 5q. *Int. J. Oncology*, **5**, 539–546.

STARZYNSKA T, BROMLEY M, GHOSH A AND STERN PL. (1992). Prognostic significance of p53 overexpression in gastric and colorectal carcinoma. *Br. J. Cancer*, **66**, 558–562.

SUN X-F, CARSTENSEN JM, ZHANG H, STAL O, WINGREN S, HATSCHEK T AND NORDENSKJOLD B. (1992). Prognostic significance of cytoplasmic p53 oncoprotein in colorectal adenocarcinoma. *Lancet*, **340**, 1369–1373.

TAKAHASHI T, NAU MM, CHIBA I, BIRRER MJ, ROSENBERG RK, VINOCOUR M, LEVITT M, PASS H, GADZAR AF AND MINNA JD. (1989). p53: a frequent target for genetic abnormalities in lung cancer. *Science*, **246**, 491–494.

TURNBULL JR, KYLE K, WATSON FR AND SPRATT J. (1967). Cancer of the colon: the influence of the no-touch isolation technic on survival rates. *Ann. Surg.*, **166**, 420–427.

VOGELSTEIN B AND KINZLER KW. (1992). p53 function and dysfunction. *Cell*, **70**, 523–526.

WARNKE R AND LEVY R. (1980). Detection of T and B cell antigens with hybridoma monoclonal antibodies. A biotin avidin horseradish peroxidase method. *J. Histochem. Cytochem.*, **28,** 771–774.

WYNFORD-THOMAS D. (1992). p53 in tumour pathology: can we trust immunocytochemistry? *J. Pathol.*, **166,** 329–330.

YAMAGUCHI A, KURAKA Y, FUSHIDA S, KANNO M, YONEMURA Y, MIWA K AND MIYAZAKI I. (1992). Expression of the p53 protein in colorectal cancer and its relationship to short term prognosis. *Cancer*, **70,** 2778–2784.

YAMAGUCHI A, NAKAGAWARA G, KUROSAKA Y, NISHIMURA G, YONEMURA Y AND MIYAZAKI I. (1993). p53 immunoreaction in endoscopic biopsy specimens of colorectal cancer, and its prognostic significance. *Br. J. Cancer*, **68,** 399–402.